# The scientific production in trauma of an emerging country

**DOI:** 10.1186/1749-7922-7-S1-S13

**Published:** 2012-08-22

**Authors:** Gustavo Pereira Fraga, Vitor Augusto de Andrade, Ricardo Schwingel, Jamil Pastori Neto, Sizenando Vieira Starling, Sandro Rizoli

**Affiliations:** 1 Division of Trauma Surgery, Department of Surgery, School of Medical Sciences, University of Campinas (Unicamp), Rua Alexander Fleming, 181 Cidade Universitária “ Prof. Zeferino Vaz ” - Barão Geraldo, Campinas - SP, Brazil; 2 School of Medical Sciences, University of Campinas (Unicamp), Campinas - SP, Brazil; 3 Hospital João XXIII, Belo Horizonte - MG, Brazil; 4 Departments of Surgery and Critical Care Medicine, Sunnybrook Health Sciences Centre, University of Toronto, Canada

## Abstract

**Background:**

The study aims to examine whether the end of specialty in trauma surgery in 2003 influenced the scientific productivity of the area in Brazil.

**Methods:**

We identified and classified the manuscripts and their authors, from databases such as *PubMed*, *Scielo* and *Plataforma Lattes* and sites like *Google*, in addition to the list of members of SBAIT, the sole society in Brazil to congregate surgeons involved in trauma care in the country. We applied statistical tests to compare the periods of 1997-2003 and 2004-2010. We also analyzed the following variables: impact factor of journals in which manuscripts were published, journals, regional origin of authors, time since graduation, and conducting post-doctorate abroad.

**Results:**

We observed a significant increase in publication rates of the analyzed groups over the years. There was a predominance of quantitative studies from the Southeast (especially the state of São Paulo). More time elapsed after graduation and the realization of postdoctoral studies abroad influenced the individual scientific productivity.

**Conclusion:**

The number of articles published by authors from the area of trauma has been growing over the past 14 years in Brazil. The end of the specialty in trauma surgery in the country did not influence the scientific productivity in the area.

## Background

Brazil is an emerging economy and a member of the “BRIC” countries, which also includes Russia, India and China. Its research labor force and research and development investment are rapidly expanding opening many new possibilities in a diversifying research portfolio. With around 85,000 papers published over a 5 year period (2003-2007), Brazil is responsible for 1.83% of the world’s papers published in journals indexed by Thomson Reuters, the agency that regularly indexes over 10,000 scientific journals worldwide [1,2].

Along with the recent economic and scientific growth of the country, the number of injuries has also grown to an astounding 130.000 deaths per year in Brazil with over 300.000 victims suffering some sequelae. Most victims of trauma in Brazil are between 5 and 14 years of age [2]. Not all is bad in Brazil that over the last decade, Brazil experienced major improvements in this scenario with the creation of stricter laws and changes in it’s traffic code leading to notable reductions in interpersonal violence and automobile crashes, which were the leading causes of death [3-7].

Despite the overall growth in trauma, in 2003 the residency training in trauma surgery during a two years program was abolished in Brazil. This change in our opinion, lead to a reduction in the number of trained professionals and academic exposure to this surgical specialty that could reduce the impetus of doing more research on the treatment of trauma disease. Therefore we hypothesized that despite the overall scientific growth in Brazil, specifically in trauma, the termination of training in trauma surgery would reduce the country scientific production in this area [8-10]. The objective of this study is to evaluate the scientific productivity in trauma, comparing the number of publications before and after the residency training in trauma was terminated in 2003 in Brazil.

## Methods

For the purpose of this study, academic production was defined as the number of publications in “trauma”. The University of Campinas (UNICAMP) Research Institutional Ethics Board approved the study and the Sociedade Brasileira de Atendimento Integrado ao Traumatizado (SBAIT) gave us consent to do the study and access to the list of all its members on December 2010.

SBAIT is the only society in Brazil to congregate surgeons dedicated to trauma care. The vast majority of the Brazilian general surgeons committed to trauma, with academic activities in trauma and holding a University appointment are members of SBAIT. It is not a governmental agency, membership is voluntary and its members are trained in general surgery and not in orthopedics or neurosurgery that congregate under the auspices of other Societies. The manuscripts published by the SBAIT members are the best sample of Brazil’s scientific production in the area of trauma.

After obtaining the list of all SBAIT members in December 2010, we identified all manuscripts they authored after 2003 (2004 to 2010). To determine whether any significant changes occurred, we performed a similar search for the same number of years, but prior to 2003, thus from 1997 to 2003. The manuscripts were retrieved from PubMed (http://www.pubmed.com), Scielo (http://www.scielo.org), the open-access online web curriculum vitae Plataforma Lattes (http://www.lattes.cnpq.br) commonly used by Brazilian investigators and a general search at Google (http://www.google.com.br).

Data collection was performed in February 2011. The manuscripts were classified as trauma when the focus was clearly on this area, or otherwise as non-trauma. For the few manuscript where the focus was uncertain, the classification was decided by consensus. The manuscripts authored by more than one SBAIT member were counted only once. Considering our goal of investigating the scientific production in Brazil, the manuscripts authored by SBAIT members that were done overseas and published in non-Brazilian journals were excluded. To evaluate the quality of the manuscripts and identify the journals favored by the Brazilian investigators, we gathered the name of the Journal, year of publication and the Impact Factor (IF) as calculated by the *Thompson Web of Knowledge* (*Institute for Scientific Information* – ISI) [11].

The first analysis aimed at studying the variations in the number of published papers before and after 2003, the year residency in trauma surgery was abolished. To this end, we tabulated the number of all publications and of all publications in trauma as well as the name of the Journals and their yearly Impact Factor since 1997. We then performed a simple comparison of the number of publications before and after 2003 and the Impact Factor of the journals.

To characterize the SBAIT members most successful in publishing in trauma, the authors were separated according to: 1. the place (state) of residence at the time of the publication; 2. the number of publications; 3. year of graduation from medical School and 4. whether they had graduate studies overseas. The year of graduation and overseas training was obtained from the open publicaly available online web CV Plataforma Lattes (http://www.lattes.cnpq.br). Next we analyzed the association between years of graduation and number of publications, as well as whether overseas training resulted in sustained increase in scientific production. The papers published during the overseas training were not included in the present analysis.

The statistical analysis used mean/median, standard deviation and maximum/minimum values for the numeric variables. The Spearman correlation was used to analyze the variation in the total number of publications, year of publication and Impact Factor. Linear regression analysis was used to estimate the association of the total number of publications, while the Mann-Whitney test was used to compare publications between the two study periods (before and after 2003). Due to the sample size and lack of normal distribution, the Kruskal-Wallis test was used to analyze time from graduation from medical School. Pearson qui-square and the exact Fisher test were used for values below 5. Significance was determined to be of 5% (p<.05) and SAS for Windows was used (version 9.1.3. SAS Institute Inc, 2002-2003, Cary, NC, USA).

## Results

In December 2010 SBAIT had a total of 320 members, which consists of the group of surgeons analyzed in the present study. Of these 320 surgeons, 104 (32.5%) published a total of 627 original papers in all areas of knowledge, of which 178 were in trauma. Considering only the work developed and published in Brazil, there were a total of 571 papers, of which 160 were in trauma. These 160 trauma papers were authored by a total of 52 surgeons, all SBAIT members.

**Figure 1:**
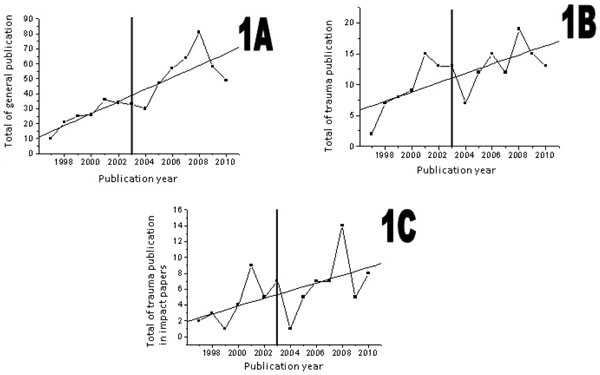
1A: Overall number of publications; 1B: number of publications in trauma;1C: number of publications in trauma in journals with any Impact Factor.

We found a significant correlation between the year of publication and the overall number of publications (r =0.89890, p = 0.001), the number of publications in trauma (r = 0, 65560, p =0.0109) as well as the number of papers in trauma published in journals with any impact factor (r = 0.60824, p =0.0210). This analysis reveals a continuing and significant increase in publication rates of the analyzed groups over the years (Figure 1). Graphs 1A (Straight regression: Y = -7995.23 +01.04 X, P <0.001), 1B (Straight regression: Y=-1494.50 + 0.75 X, P = 0.004) and 1C (Straight regression: Y=-71.96 00:49 + X, P = 0.029) disclose the linear regression analysis and the association between the year of publication and total number of publications and the trend towards an increased number of publications.

The comparative analysis between the periods before (1997 to 2003) and after 2003 (2004 to 2010) showed a statistically significant difference only on the overall number of publications, which was higher after 2003 (p = 0.006). The total number of publications in trauma (p = 0.196) and trauma in journals with impact factor (p = 0.245) was not statistically different. No statistically significant difference was found on the year of publication and impact factor of journals published (p = 0.3683), the study of linear trend between years and the impact factor by linear regression (p = 0.510) and comparison of the impact factor among two periods (p = 0.477).

Table 1 show the list of top 10 journals in the world that have published Brazilian papers in trauma.

**Table 1:** List of top 10 journals that have published Brazilian papers in trauma.

Journal	Number of papers
Revista do Colégio Brasileiro de Cirurgiões	54
Journal of Trauma	16
Revista da Associação Medica Brasileira	15
Acta Cirúrgica Brasileira	12
Injury - International Journal of the Care of the Injured	7
Revista do Hospital das Clínicas	6
World Journal of Emergency Surgery	4
Revista de Saúde Pública	3
Jornal Vascular Brasileiro	3
Sao Paulo Medical Journal	3

Table 2 shows the regional distribution of the SBAIT members with publications in trauma between 1997 and 2010.

**Table 2:** SBAIT member distributions by region and publication.

Region	Total of members	Published	Published on trauma
Southeast	160	66	35
Northeast	64	11	4
South	46	16	9
North	37	8	4
Midwest	13	3	0

**Figure 2:**
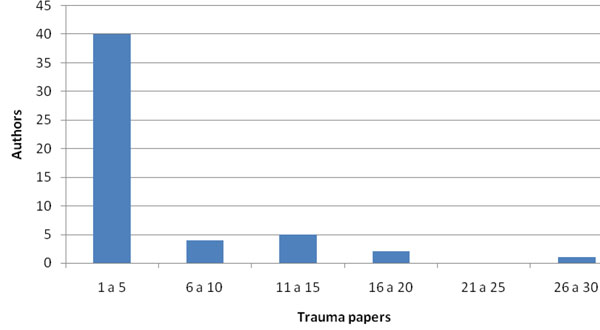
Number of papers in trauma per authors.

The Southeastern region of Brazil had 160 surgeons that were members of SBAIT in December 2010. Of these, 101 were from Sao Paulo state, 45 had published at least 1 paper and 30 had authored papers in trauma. Sao Paulo state had the highest number of publications in Brazil. Compared to the other states, Sao Paulo had significantly more SBAIT members with publications (p =0.002) and more publications per author in trauma (p = 0.003). When the two periods were compared, the number of publications from Sao Paulo continued to be significantly higher (p = 0.003). Of the 160 papers published, 52 were authored by surgeons from Sao Paulo. The same was observed with trauma publications authored by 30 (57.7%) surgeons from the State of Sao Paulo. About ¼ of the authors from Sao Paulo (12 or 23%) published more than five papers in this period. Figure 2 shows the distribution of the 52 authors by number of papers published in trauma.

The number of years from graduation from medical school of the 104 SBAIT members authoring papers in Brazil on all topics over the study period was of 22.4 years, varying from 1 to 49 years. Table 3 shows the number of years since graduation for the 104 authors. Statistical analysis revealed significant correlation between the elapsed time after graduation and the number of publications of each author in trauma, the authors show that with more time graduation held the largest number of published studies (p =0.0373).

**Table 3:** Number of years from graduation from medical schools and number of publications.

Time of graduation	Number of authors	Average general publications	Average numbers of publications in trauma
< 5 years	5	2,2	0,6
6 – 10 years	11	2,2	0,3
11 – 15 years	6	1,3	0,7
16 – 20 years	23	10,9	3,6
21 – 25 years	18	3,6	1,4
26 – 30 years	19	8,6	2,0
31 – 35 years	14	7,8	1,6
> 35 years	8	23,8	8,9

Of the 320 SBAIT members in December 2010, 10 had post-doctoral training overseas: 6 in the United States, 1 in Canada, 1 in both the United States and Canada, 1 in France and 1 in Germany. There was a significant difference between the number of publications by these 10 surgeons and the 94 other ones on the number of publications in Brazil and overseas (p <0.001; p <0.001 respectively) (Table 4).

**Table 4:** SBAIT members with post-doctoral training overseas and number of publications.

Post doctorate at exterior	Number of years since graduation	Number of papers published during overseas post-doctoral training	Number of papers published in Brazil
No = 94	1 – 49 (Average: 22,2)	-	1 - 56 (Total: 520; Average: 5,6)
Yes = 10	17 - 33 (Average: 24,3)	0 - 6 (Total: 17; Average: 3,4)	3 - 86 (Total: 249; Average: 24,9)

## Discussion

This study is important because is the first to examine the scientific contribution of an emerging country in trauma. Overall the number of publications undertaken and supported by Brazilian continuously grew over the last 14 years (Figure 1A, 1BA, 1C). This increase, demonstrated in Figure 1A, paralleled the trend in scientific production in surgery over the last decade demonstrated by Heldwein et al [2]. Possible explanations for this increase may be inputed to increasing funding for research by the Brazilian government, particularly the Ministry of Health that over the last decade increased the opportunities for international exchange and dissemination of Internet use [2,12,13].

The number of publications devoted to trauma, analyzed as a whole and also in relation to the proportion published in journals with impact factor, followed the increased productivity of Brazilian researchers, showing that the production has grown not only in absolute numbers, but also in quality [2,14]. Thus, the end of residency in trauma surgery in Brazil did not seem to have affected the scientific development of the area nor the enthusiasm of the authors [8,9,15]. The sustained growth may be explained by the greater diffusion of courses such as the Advanced Trauma Life Support (ATLS) and scientific events throughout the country, which also grew enormously over the last decade (results not shown). We consider that the greater involvement of professionals in trauma is very welcome in our country, given the increasing numbers of motor vehicle collisions and domestic violence. According to the Information System (SIM), which collects national data, the period comprising the years 1998 and 2008, the total number of homicides rose from 41,950 to 50,113 (an increase of 17.8%, higher than the population growth of 17.2% over the same period, despite the disarmament policies developed mainly from 2004), and deaths from traffic crashes increased from 30,994 to 39,211 (an increase of 20.8%, also higher population growth, despite the enactment of the last Traffic Code in 1997 which led to a decrease in the quantity of violence, but in absolute terms, lasted only three years - 1997 to 2000) [4,6,7,16-19].

In this study, we chose not to analyze the quality of studies, which could be done by analyzing the number of times they were actually cited. We still performed an evaluation of the quality when we analyzed the impact factor of the journals that published the studies. We opted for the impact factor, since it provides a global assessment of the insertion of Brazilian investigators in the national and international setting of scientific publications. It is important to mention that no single parameters is ideal for determining the quality of publications since high-impact journals can still publish low impact studies [16,20].

Zhi Li et al. [20] analyzed the characteristics of publications in urgent and emergency care by Chinese authors. He reported that over a period of 10 years 932 studies were published and the number of publications grew over the years. The Journal of Trauma was used the most by the authors surveyed. When he analyzed the 18 major journals specialized in trauma, this author found that the United States (from 1999 to 2008) was the country with the highest number of publication in trauma with 9956 articles. It was followed by Germany, Britain, France and Japan, with 2668, 2460, 1301 and 998 publications each. Despite many major differences that which prevent a reasonable comparison, our study shows that Brazilian surgeons published less than the countries described above with 160 publications in 38 journals. However we must also consider that significant social, cultural, economical and scientific differences between Brazil and the other countries. Under this perspective, we think that the number of publications by Brazilian surgeons is encouraging particularly when one considers the continuous growth remains significant, especially considering the scientific context of the country.

The Journal of the Brazilian College of Surgeons (Revista do Colegio Brasileiro de Cirurgioes) was the journal with the largest number of publications by Brazilian surgeons including trauma papers. The JBCS is published bimonthly and was founded in 1930 by the Brazilian College of Surgeons. The non-Brazilian with the largest number of publications was the Journal of Trauma, founded in 1961 and specialized in trauma and emergency surgery (Table 1). In the Chinese study by Zhi Li et al. [20] the Journal of Trauma was also the one that published most Chinese papers.

The southeast region of Brazil has the highest population density in the country, housing 42% of the Brazilian population. The State of São Paulo alone is home to about 50% of all the southeast population and 55% of all the SBAIT members living in the southeast region. Sao Paulo has the largest Gross Domestic Product (GDP) of the country [21,22], the largest vehicle fleet and rate of urbanization, all social factors that are directly related to the leading causes of death from trauma: motor vehicle collisions and homicides [3]. The southeast has five of the largest universities in the country resulting in the State of Sao Paulo alone producing 38% of all Brazilian publications and in 2008, 1.83% of all publications in the world [1,2,13].

Our results demonstrate that after Sao Paulo Minas Gerais, Rio Grande do Sul and Parana are the ones with the largest number of publications in general surgery.

Despite the observed growth in research we observed, the number of publications being done in Brazil remain small [1,23]. The Coordination for the Improvement of Higher Education Personnel (CAPES) recently reported that more than 50% of all dissertations and doctoral thesis made in Brazil are not published, a fact that can make the country invisible to the scientific community [24,25].

Another interesting finding of the present study is that ¾ of the authors (76.9%) published up to five papers, 1/6 (17.3%) 6 to 20 papers and 1/20 (5.76%) published 26 to 30 papers. The group of surgeons that were SBAIT members in 2010 that published studies is small but considerable 16.3%. Seniority, measured in this study as years from graduation, correlated with scientific productivity: those with the highest number of publications were also the most seniors, especially those with more than 35 years since graduation. If 3 of the 8 investigators with more than 35 years since graduation were excluded from the analysis, the average number of publications would be much lower (7.2 for all publications and 1.4 for trauma) and the most productive group would consist of those between 16 and 20 years of graduation.

Our study also shows the significant and positive influence of post-doctoral training overseas on scientific publications. Of the 104 authors, only 10 had post doctoral training overseas but their a average number of publications was 4.4 times higher than the others. These results are in agreement with the work of other authors [24,25]. Such training foster collaboration between institutions and investigators and reinforce the importance of promoting such training, promoting cooperation between institutions, evolution of organizations, and development of scientific production [24,25].

## Conclusion

The number of papers published in Brazilian journals by Brazilian surgeons in surgery and trauma has experienced a linear growth over the past 14 years. We were unable to identify any evidence that the end of residency in trauma surgery in Brazil negatively influenced the scientific production in this area. The main characteristics of the Brazilian surgeons that write papers in trauma can be described as someone that lives in the southeast of Brazil, most likely in the State of São Paulo and graduated from medical school more than 16 years ago. The observed growth in the number of publications parallels the economic growth of the country and the investments made by the Brazilian government in research and development over recent years. New possibilities of research in this area of knowledge can be offered, with options for expansion of partnership and international cooperation for the development of science. Our study suggests that the scientific growth in this specific area of surgery (trauma) is more likely the result of an overall growth in research and development and less due to specific growth in trauma as can be attested by the fact that the end of the residency program in trauma surgery in 2003 had no apparent effect in the number of publications in trauma.

## Competing interests

None.

## Authors’ contributions

GPF had overall responsibility for the study including conception, design and intellectual content, collection, analysis and interpretation of data. VAdA participated in the conception, design and intellectual content, collection, analysis and interpretation of data. RS participated in the conception, design and intellectual content, collection, analysis and interpretation of data. JPN participated in the conception, design and intellectual content, collection, analysis and interpretation of data. SVS participated in the intellectual content, revision of the manuscript, figures and tables. SR participated in the intellectual content, revision of the manuscript, figures and tables.

## References

[B1] Country Profiles: 2009: Top 20 Countries in ALL FIELDS, 1999- August 31, 2009 Avaible at: http://sciencewatch.com/dr/cou/pdf/09decALL.pdf.

[B2] Heldwein FL Hartmann AA Kalil AN Neves BVD Ratti GSB Beber MC Jr Cited Brazilian papers in general surgery between 1970 and 2009 Clinics 2010 65 5 521 529 10.1590/S1807-59322010000500010 20535371 PMC2882547

[B3] Waiselfisz JJ Map of Violence 2011. The young people of Brazil Brasília: Ministry of Justice 2009

[B4] Reichenheim ME Souza ER Moraes CL Jorge MHPM Silva CMFP Minaya MCS Violence and injuries in Brazil: the effect, progress made, and challenges ahead Lancet 2011 377 1962 1975 10.1016/S0140-6736(11)60053-6 21561649

[B5] Paim J Travassos C Almeida C Bahia L Macinko J The Brazilian health system: history, advances, and challenges Lancet 2011 377 1778 1797 10.1016/S0140-6736(11)60054-8 21561655

[B6] Victora GC Barreto ML Leal MC Monteiro CA Schmidt MI Paim J Health conditions and health-policy innovations in Brazil: the way forward Lancet 2011 377 2042 2053 10.1016/S0140-6736(11)60055-X 21561659

[B7] Almeida-Filho A Higher education and health care in Brazil Lancet 2011 377 1898 1900 10.1016/S0140-6736(11)60326-7 21561653

[B8] Birolini D Trauma: social and medical challenge J Am Coll Surg 2008 207 1 1 6 10.1016/j.jamcollsurg.2008.02.005 18589354

[B9] Green SM Trauma surgery: discipline in crisis Ann Emerg Med 2009 53 198 207 10.1016/j.annemergmed.2008.03.023 18439724

[B10] The Committee to Development the Reorganized Specialty of Trauma, Surgical Critical Care, and Emergency Surgery Acute Care Surgery: Trauma, Critical care, and Emergency Surgery J Trauma 2005 58 614 616 15761359 10.1097/01.ta.0000159347.03278.e1

[B11] ISI Web of knowledge database Available at: http://apps.isiknowledge.com.

[B12] Ministry of Health Department of Science and Technology, Ministry of Science, Technology and Strategic Inputs Decentralization in the context of promoting health research Rev. Saúde Pública 2011 45 3 626 630 10.1590/s0034-89102011000300023 21552761

[B13] Marques F Advances and challenges Fapesp 2011 185 26 33

[B14] Berwanger O Riberio RA Finkelsztejn A Watanabe M Suzumura EA Duncan BB The quality of reporting of trial abstracts is suboptimal: Survey of major general medical journals Journal of Clinical Epidemiology 2009 62 387 392 10.1016/j.jclinepi.2008.05.013 19010643

[B15] Ciesla DJ Moore EE Moore JB Johnson JL Cothren CC Burch JM The Academic Trauma Center Is a Model for the Future Trauma and Acute Care Surgeon J.Trauma 2005 58 4 657 662 10.1097/01.TA.0000159241.62333.94 15824639

[B16] Schimidt MI Duncan BB Silva GA Menezes AN Monteiro AC Barreto SM Chronic non-communicable diseases in Brazil: burden and current challenges Lancet 2011 377 1949 1961 10.1016/S0140-6736(11)60135-9 21561658

[B17] Mello Jorge M Koizumi M Traffic accidents in Brazil: an atlas of their distribution São Paulo 2007 ABRAMET

[B18] Krug EG Dahlberg LL Mercy JA Zwi AB Lozano R World report on violence and health 2002 Geneva: World Health Organization

[B19] WHO Age-standardized mortality rates by cause (per 100 000 population) 2008 Geneva: World Health Organization Avaible at: http://www.who.int/whosis/indicators/compendium/2008/1mst/en/index.htm.

[B20] Li Z Liao Z Wu FX Yang LQ Sun YM Yu WF Scientific publications in critical care medicine journals from Chinese authors: a 10-year survey of the literature J Trauma 2010 69 4 E20 3 10.1097/TA.0b013e3181c45257 20179654

[B21] BRAZIL. Ministry of Planning, Budget and Management. Brazilian Institute of Geography and Statistics Population Count Available at: http://www.censo2010.ibge.gov.br.

[B22] BRAZIL. Ministry of Planning, Budget and Management. Brazilian Institute of Geography and Statistics Population Count Available at:http://www.ibge.gov.br/home/download/estatistica.shtm

[B23] Andrade VA Carpini S Schwingel R Calderan Fraga GP Publication of papers presented in a Brazilian Trauma Congress Rev Col Bras Cir 2011 38 3 172 176 10.1590/S0100-69912011000300006 21789455

[B24] Castro PMR Porto GS Return abroad worth it? The issue of post-doctoral stages from the perspective of production in S & T Organizations & Society 2008 15 47 155 173

[B25] Calvosa MVD Repossi MG Castro PMR Evaluation results of teacher training: post-doctoral fellow at Universidade Federal Fluminense in light of scientific and literature Rating (Campinas), Sorocaba 2011 16 1 99 122

